# *“The others look at you as if you are a grave”*: a qualitative study of subjective experiences of patients with epilepsy regarding their treatment and care in Cape Town, South Africa

**DOI:** 10.1186/s12914-016-0084-0

**Published:** 2016-03-17

**Authors:** Mpoe Johannah Keikelame, Leslie Swartz

**Affiliations:** Primary Health Care Directorate, Faculty of Health Sciences, University of Cape Town, Cape Town, South Africa; Department of Psychology, Stellenbosch University, Cape Town, South Africa

**Keywords:** Patients with epilepsy, Treatment and care, Routine task system, Qualitative study, South Africa

## Abstract

**Background:**

Existing evidence shows that the majority of people with epilepsy in lower and middle income countries are not receiving appropriate treatment and care. Although this problem has been reported as one of the factors affecting the quality of lives of people with epilepsy, very few studies have investigated patients’ perspectives and their experiences about the problem. This qualitative study explored perspectives and subjective experiences of people with epilepsy about their illness in an urban township in South Africa.

**Methods:**

Individual face-to-face interviews included twelve people who had epilepsy. A semi-structured interview guide which was based on Kleinman (1980) Explanatory Models Framework was used to elicit participants’ perspectives and subjective experiences about their illness and its treatment. Thematic analysis method was used to analyse the data.

**Results:**

The main theme reflecting participants’ verbatim accounts was about their perceived difficulties affecting their access to treatment and care during their routine clinical follow up visits. These concerned rushed consultations which focussed on seizure frequency and adherence to medication with no attention to personal concerns. They perceived that part of the problem could be that some health care practitioners were not adequately trained and lacked empathy, interest, respect and listening skills. We argue that in a health system where patients feel that they are not respected and their concerns are not listened to or are ignored, they may lack trust in the system and this may violate their right to access treatment and care.

**Conclusions:**

The findings provide a glimpse of the extent to which the power and nature of the routine task-centred system can lead to violation of patients’ health rights – especially with epilepsy which is poorly understood and stigmatized. Appropriate interventions are needed to address health system factors affecting the treatment and care of this marginalized and vulnerable group of patients.

## Background

The importance of improving the quality of care for patients living with chronic illnesses is reported as a global concern [1, 2]. With respect to epilepsy, this is of particular importance because various reports highlight that most patients with epilepsy (PWE) in low and middle income countries (LMICs) are not receiving appropriate treatment and care [3, 4]. Epilepsy has been reported globally as one of the most common neurological conditions [5] which contributes to approximately 1 % of the global burden of disease [6, 7] and 20 % thereof is in Africa [8]. In LMICs per se, the provision of epilepsy treatment and care is characterized by inequalities between the public and private health care sectors [9] and a lack of adequate financial resources to address the treatment gap related factors [10]. What is of great concern is that neurological conditions in LMICs are commonly treated within the mental health sector which is described as “one of the notoriously underfunded sector” (p. 180) [5], with the median percentage of health expenditure estimated at 0.5 % in LMICs and 5.1 % in high income countries [5].

In response to the need for the improvement of epilepsy care in LMICs, evidence-based guidelines have been developed by the World Health Organization's (WHO) mental health Gap Action Program (mhGAP), but these need to be adapted and tailored for appropriate implementation in respective countries [5, 11]. Although the mhGAP puts emphasis on protection and promotion of human rights as well as the provision of community based care and support [12], it has been reported to lack focus on the social and cultural factors affecting mental health promotion [13]. This is crucial especially with a condition such as epilepsy which is rooted in superstitious beliefs about its cause and treatment [14]. While there are indicators that have been set to assess and monitor the effectiveness and appropriateness of these epilepsy guidelines [15–17], Varley, Delanty, Normand et al. [18] and Fitzsimons, Normand, Varley et al. [19] state that they do not show any major improvements in the quality of care of PWE.

In South Africa, PWE, like patients with other chronic illnesses, receive their treatment and care from the public health sector which serves most of the low socio-economic population groups [20]. While access to appropriate care is a constitutional right [21], inequitable resource allocation remains a big problem [21, 22]. With regard to epilepsy, the situation could be worse than with other chronic illnesses. First, epilepsy is perceived to be poorly managed in primary care settings in Cape Town [23]. Second, clinical audits are conducted for other chronic illnesses (asthma, hypertension, Chronic Obstructive Pulmonary Diseases (COPDs) as well as epilepsy – however epilepsy is only reported as a new area for clinical audits [24]. Third, there is also a lack of accurate statistics on the burden of epilepsy [25] probably because epilepsy statistics are combined with other neurological conditions [26].

In this article, we present findings from twelve individual interviews with PWE as part of a larger study which explored perspectives on epilepsy on the part of patients and carers in an urban township in Cape Town, South Africa. Our research question was: How do PWE in an urban township in Cape Town understand the illness (epilepsy)?

Our study aim was to analyse and describe the subjective experiences and perspectives of PWE. The objective was to provide information that can be used to guide future policy, planning and development of appropriate interventions to address treatment and care challenges faced by PWE.

## Methods

### Research design

To explore subjective experiences of PWE and their perspectives on their illness, we used a qualitative design because it is flexible and emergent and uses a naturalistic approach [27–29].

### Study setting

The study setting is one of the oldest urban townships in Cape Town which was developed as a result of the past laws of the oppressive colonial and apartheid rule. The predominant spoken language is isiXhosa – but there are some other African languages such as Sesotho and isiZulu. Most of the residents receive health care from the local Community Health Centre (CHC) which serves the Afrikaans and Xhosa speaking communities. The setting is characterized by poverty, unemployment, illiteracy, crime, lack of proper housing, sanitation and water [30]. There is a local clinic that offers preventative health care services such as child health and reproductive health, Tuberculosis (TB), Human Immunodeficiency Virus (HIV) and Acquired Immune Deficiency Syndrome (AIDS). Other health care services are provided by private general practitioners, traditional healers and non-governmental organisations (NGOs) [30]. Although epilepsy support services for patients and family members are available, they are currently inaccessible to the study population.

### Recruitment and access to participants

Recruitment and access to participants was done by MJK after approval of the project by the four local ethics committees: the Health Sciences Research Ethics Committee, University of Cape Town; the Health Sciences Ethics Committee, Stellenbosch University; the Provincial Government of the Western Cape (PGWC) and City Health. Prior to recruitment, MJK presented a summary of the study background and its aims and objectives to the local health committee and the traditional healer’s organisation (THO) in order to gain support – this has been highlighted by Mack et al. [31] as an important strategy. From these meetings with the local gate keepers, additional potential PWE who were not receiving treatment and care from the local community health centre (CHC) where MJK had initially recruited were identified. Others were identified by field assistants who were doing volunteer work in the study setting and were all included in the list. We used purposive and snowball sampling methods because they fitted our inclusion criteria and the research question [31]. Our criteria stated that only Xhosa speaking PWE who were aged 18 years and older and resided in the study setting would be included.

A total of 24 potential PWE were listed and were visited by MJK at their homes to formally recruit them. Only 12 PWE agreed to participate in the study. They were all visited by MJK at their homes to formally obtain written informed consent. The informed consent forms and participant information leaflets were translated into their local language, isiXhosa (one of the predominant languages spoken in Cape Town, more commonly referred to as “Xhosa”). In the process, MJK was assisted by two Xhosa speaking field assistants to interpret the information leaflets because, although she is conversant in isiXhosa, she would not be able to read the Xhosa dialects as fluently as would a native speaker. All participants were each given their signed copies of the consent forms and were given time to acquaint themselves with the information and to discuss the documents with family members and friends to legitimize their understanding of the purpose of the study and their rights and responsibilities in the process.

### Data collection

Data collection was done by MJK between September 2012 and July 2013 at the participants’ homes on the agreed upon date and time. A semi-structured interview guide which was translated into isiXhosa was piloted and was used to collect the data. No changes were made after piloting. The instrument consisted of open-ended questions based on Kleinman (1980) EMs framework and elicited qualitative responses on participants’ explanations of epilepsy in terms of what they call the illness; what they think caused their illness (epilepsy); why they think it started when it did; what they think the illness does and how it works; how severe their illness is and whether it will have a short or long course; the kind of treatment they think they should have and the expected treatment outcomes; the main difficulties caused by the illness at different levels (individual, family, community, health system and society) and what they feared most about the illness [32, 33]. According to Kleinman (1980), EMs are ways of understanding how people recognize an illness, explain it and respond to it and are also shaped and influenced by culture and are held by patients and carers. In addition, EMs can also enable a deeper understanding of what it means to suffer from an illness in the context of the person having the illness, his or her family, community and the health care system [34].

Prior to conducting individual in-depth face to face interviews, MJK explained the purpose of the interview and how anonymity and confidentiality would be kept, including how participants’ information would be disseminated. All interviews were audio-recorded with participants’ written permission and were all conducted in the participants’ home language, isiXhosa. The duration of the interviews was between 45 and 90 min. At the end of each interview, MJK wrote field notes immediately and these were later extended after reflection [31] – and were used by MJK in reflective meetings with the second author (LS) to gain an understanding of what participants had said. Participants were each given a R60.00 (approximately $6) food voucher after the interview as a token of appreciation for their time and contribution.

### Data analysis

The audio-recorded data were transcribed verbatim from isiXhosa into English by a Xhosa speaking transcriptionist. MJK designed a confidential transcription agreement to ensure that the transcriber maintained confidentiality, privacy and storage of the data during the transcription process. A signed copy was kept for record keeping by MJK. Consistent with Braun and Clarke’s [35] thematic analysis method, MJK read all transcripts and listened to each individual audio-recorded interview to familiarize herself with the data and to ensure that the transcribed data captured the actual verbatim responses expressed by all participants. This process of re-reading transcripts and listening to the audio-recorded data enables the investigator to be immersed in the data and to become familiar with it [35]. Using an inductive approach [35], MJK read each transcript, noted ideas and made notes generated through familiarization and immersion. All data were coded by MJK and during open coding, data driven codes and themes were generated, reviewed, defined, described and named to provide a clear sense of what each theme is about. Thereafter, MJK organized the main key themes that emerged from the interview data into tables and grouped them into appropriate categories with quotations from transcripts and compared them for similarities and differences. MJK modified, grouped and re-grouped codes to ensure no codes have been missed in the earlier stages of the analysis and that they are grounded in the data [35].

MJK and LS had regular feedback and debriefing meetings, and discussed, compared and contrasted the coded data to increase rigor and to gain consensus [36, 37]. Thematic maps were drawn by MJK to show links and relationships between the themes, these themes being further organized into sub-themes which had emerged from each theme. These were used by MJK and LS to further undertake a rigorous systematic analysis of the individual transcripts and thematic maps in order to search for alternative explanations [35]. MJK made appointments with all interviewees and visited them at their homes to present the draft findings and to confirm interpretation thereof [36, 38].

## Results

### Participants’ characteristics

Twelve PWE were individually interviewed. Eight were males and four were females. The mean age for men was 47 years, and 37 years for females. All participants were Xhosa speaking and had been residing in the study setting for more than 20 years. All had attended school but only two had passed grade 10. The mean number of years of living with epilepsy for males was 29.9 years and 27.2 years for females. Three males were married and one lived with a partner. Only one female was married and two were youth (aged 25 and 28 years). In South Africa, the National Development Youth Agency (NYDA) Policy [39] defines people who are aged between 14 and 35 years as youth. All participants were on seizure medication and some informed us that besides being on seizure medication, they were also receiving treatment for other conditions such as asthma and hypertension and only one reported being on Antiretroviral (ARV) treatment. Six were receiving a government disability grant as a source of income and one was receiving an old age pension grant. Three stated that they had applied for a disability grant on several occasions, but their application had been declined. Only two males were in part-time employment.

**Fig. 1:**
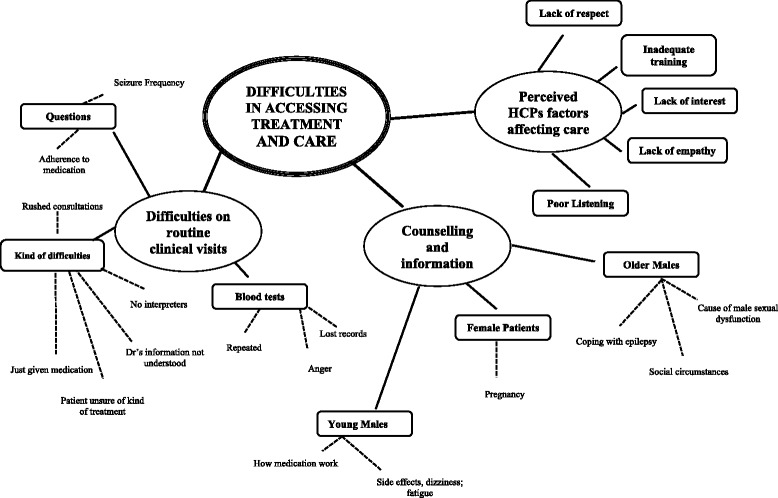
Schematic of the theme ‘Difficulties in accessing treatment and care’ including sub-themes and examples of perceived factors reported by respondents

Seven key themes emerged from the data: (i) names referring to epilepsy; (ii) views about the illness; (iii) beliefs about the cause of the illness; (iv) views about the kind of treatment for epilepsy; (v) views about marriage, driving, employment and schooling; (vi) difficulties in accessing treatment and care; and (vii) fears about the illness. We report here on one key theme: “difficulties in accessing treatment and care” which is one of the key issues that all participants talked about in their interviews. We also provide a thematic map in Fig. 1 which shows this main key theme and its three sub-themes which emerged from the data: (i) difficulties on routine clinical visits; (ii) perceived health care practitioners’ (HCPs) factors affecting care; and (iii) counselling and information needs.

### Theme: difficulties in accessing treatment and care

Participants were asked about the kind of difficulties they experience when seeking treatment and care. Their responses are discussed below under the following three sub-themes:

#### Difficulties on routine clinical visits

The respondents’ provided varied explanations of the kind of difficulties that inhibit their access to appropriate treatment and care on routine clinical visits. Two male participants said that the clinical examinations focus on seizure frequency and adherence to medication:

*On my doctor’s date, the doctor checks and asks when I last had fits… I explain that I last did* [had a seizure] *at a certain time. He’d say, “No, you’ll just have to fetch pills…” There is nothing that they ask…* [Male participant aged 62]

*When you get there* [at the clinic], *you are asked when were you last sick* [when you last had a seizure]…*how much time has elapsed since you were sick…do you use the treatment…that’s it. When you are done with they just sign whatever it is that they sign, you don’t know what it is, and then they say go and get your pills, and that is all… You are not told anything…* [Male participant aged 45]

The same male participant explained that he had difficulties in comprehending doctors’ explanations about the kind of surgical treatment that could be done for his seizures. He was of the view that such information can only be understood by people who are educated and who are also able to interact with doctors:

*When I went to a doctor* [western trained] *here… I don’t know what he said…that my condition could be cured…but it sounded like there’s something that needs to be done to me…something like an operation in the head… I didn’t know what he meant. You see, things like that need people who are educated who will be able to ask him questions the way that he talks…* [Male participant aged 45]

This same male participant further highlighted that the situation at the CHC is very difficult because consultations are rushed with no time to listen to patients’ concerns:

*The thing at CHC is not really easy, because they are in a hurry… You can explain an issue of concern* [problem] *many times, you can tell them today, tell them on the following month, and tell them again the following month*… [Male participant aged 45]

Another male participant provided a detailed explanation of how he was particularly angered and distressed by being informed on his routine follow up visit that his blood results could not be found. He was therefore subjected to have the blood test repeated despite this being against his will:

*They* [HCPs] *drew blood last year on the 13th of September, and they told me to wait, maybe two weeks and then go and check… I did that. When I went there to get my results, they couldn’t find them. They told me to go to the doctor… The doctor wrote something down… They read it for me and said that the doctor says that you should have blood drawn. I asked why because I had blood drawn the week before last, now why is it getting drawn? They said he wants to see about the pills that I was given to take. They took it even though I didn’t want to… I was angry about that…* [Male participant aged 28]

This participant further explained that lack of interpreters to support Xhosa patients and doctors who are not proficient in isiXhosa affects patient and doctor interaction:

*The difficulty there is that…you as a Black person have no one to speak for you because there are White doctors… They don’t speak Xhosa…we don’t know what they are saying…* [Male participant aged 28]

#### Perceived HCPs factors affecting care

Some respondents were critical of the way care is provided by some HCPs. According to them, factors such as lack of empathy, lack of respect, passive and poor listening, lack of interest and poor training of nurses affected their treatment and care.

One male participant was critical of the politics of the past oppressive health system and thought those who had experienced such injustices would be in the best position to render better care – but instead, it is the White doctors and nurses who provide better care:

*The nurses that are Black and Coloured, they don’t care about us…they forget that they are here because of us. They don’t care about us no matter that we come from White oppression…but the Whites who play that role of doctors and nurses…they care more than our Black people and the Coloureds.* [Male participant aged 45]

The same participant further elaborated that there are some health practitioners who are disrespectful and often do not provide information required by patients:

*You see the others look at you as if you are a grave…they just glance at you…even when you talk to her, she says, “I am busy,” as if you don’t know that. You are just asking something that you don’t know…she is the one who knows…she won’t even want to listen… Those things bother us…* [Male participant aged 45]

One female participant thought that doctors are passive listeners and are therefore unable to interpret what patients say and this may affect the doctors’ approach to treatment and care:

*Sometimes it’s like the doctors also have a problem of not hearing…you tell him everything… He doesn’t hear whilst he is listening. So it is a problem there to him… Now when he doesn’t hear you… The big problem you went for to the clinic didn’t get attention… You find that you just went, you come out having stress…* [Female participant aged 42]

Two male respondents were of the view that some doctors are not interested in discussing patients’ concerns. One said that he informed the doctor about the fatigue and dizziness that he experiences from taking seizure medication, but the doctor said that those symptoms are common. The other one said that he had been informing HCPs about his sexual health problem – but he has lost hope because they did not care about it.

*Since I started seeing the doctors it’s as if they are not interested. That’s the way I see it… I tell the doctor about my fatigue…but I don’t know if they take note of that or if they don’t. Another one said, when I told him I feel weak. I just become dizzy sometimes when I take these tablets… He said that it is something that is common*… [Male participant aged 42]

*For long, I told them about my manhood problem… I have since given up on them… They did not care*… [Male participant aged 62]

Another female respondent reported that she informed the doctor about her difficulties with regard to how she copes with her illness, but it seemed the doctor was not interested in what she was telling him:

*When I arrive there at the doctor…I tell him everything about my situation that it is this way and that way…but he likes to cut me… He doesn’t listen to how I go* [doctor does not listen to how she copes with the illness]*…* [Female participant aged 53]

#### Counselling and information needs

We asked our participants about the issues that they want to discuss with their HCPs on their routine clinical visits. We found that their individual needs were varied and were based on their age and gender – and included information needs on seizure medication and reasons for experiencing certain side effects, male sexual health problems and pregnancy. Two male participants said that they would like to be informed about why the seizure medication causes them to become dizzy:

*I would like them to explain to me why for such a long time since I started medication in 2006…I am still feeling dizzy when I take these pills and I have no strength… I would really like that they explain about why the medication doesn’t get used to me…* [Male participant aged 42]

*I have been taking these medicines since I was young… I noticed this thing that every time I take these pills I feel dizzy… I would feel like someone who had taken an alcoholic drink and I don’t even touch that… I want to know why I don’t get better*… [Male participant aged 28]

An older male participant wanted to know the cause of his sexual dysfunction:

*I wanted to know what causes that if I want to meet with her* [has sexual desire for his wife]…*then the lights turn off first very quickly* [has difficulty in maintaining an erection]…*it wasn’t like this before…* [Male participant aged 62]

It was interesting to note that one female participant who was of child-bearing age said that she was interested to know about pregnancy related issues in epilepsy but the doctors’ response was insufficient:

*I asked the doctor if a person with epilepsy* [meaning a female person who has epilepsy] *can get pregnant. That doctor said yes and it ended there…* [Female participant aged 25]

A male participant who had recently been diagnosed with epilepsy said that he wished to be counselled on how to cope with the illness and the kind of treatment that can help to achieve seizure freedom:

*I wish they could ask me how I am coping with this illness*… *I am troubled by this illness… It is because when you are not well and being held by an illness…you’re always not happy because I’m always busy thinking of how I can get a plan that can free me from my problem* [meaning his illness]*…* [Male participant aged 56]

Another male participant said that he wished to be counselled on his socio-economic circumstances:

*I wish they could ask me how I sit here at home, what I eat because I don’t work. Even when I ask for the grant they just say I haven’t reached 60*… [Male participant aged 58]

## Discussion

Our qualitative analysis of our participants’ explanations of the difficulties affecting their treatment and care provided a glimpse of experiences of twelve patients who have been living with epilepsy for more than twenty years. Most participants were uneducated and unemployed and very few had a reliable income. The participants’ experiences on their routine clinical visits show that they have in fact experienced inappropriate treatment and care and that their individual needs or concerns were not attended to. Their verbatim accounts provide some insights on the kind of health system factors that may lead to violation of patients’ health rights. Some examples of the types and nature of these violations have been reported by Vivian et al. [40] in health care settings in South Africa.

Our respondents’ descriptions of their experiences of treatment and care during routine clinical visits confirm findings from the observational study of PWE conducted by Keikelame and Swartz [41] in a chronic illness clinic in Cape Town. According to Rubin et al. [42], positive health or treatment outcomes occur where patients are empowered to ask questions as opposed to questions being asked by HCPs. In our study, some participants stated that they asked questions and highlighted issues of personal concern that they wanted to discuss with HCPs – such as reproductive and gender related issues (male sexual health problems and pregnancy), socio-economic circumstances and how they cope with the illness – but these were either ignored or seen as unimportant. They also stated that they were only asked questions on frequency of seizures and adherence to their seizure medication – yet there were some who reported that they had other chronic illnesses. This finding is important since comorbidities in patients with non-communicable diseases (NCDs) have been reported to pose serious challenges for HCPs in South African primary care settings [43]. While there was only one participant who reported being on ARV treatment, the finding is of great concern because of drug interactions when anti-epileptic drugs (AEDs) and ARVs are co-administered [44]. These findings suggest a need for an integrated approach to management of NCDs in general.

There were some health systems factors which were perceived to inhibit patients’ access to appropriate treatment and care which occurred through the routine task-centred system (RTCS) which Van der Walt [45] defines as a “compartmentalized system which seems to offer a degree of emotional protection to the nurses in their dealings with patients” (p. 77). The author further states that this protection seems to be a powerful means through which patients are subjected to care and which focuses on tasks rather than on engaging with the experience of illness. This type of system may lead to disempowerment of patients and may also inhibit HCPs to provide appropriate treatment and care. Writing about the influence of power on patient care, Powell and Davies [46] state that power has a great influence on patient care, especially in complex environments where it is provided by different HCPs who have their own cultures, identities, and educational backgrounds – and who are also very complex and divided [47]. Furthermore, Doherty and Stavropoulou [48] are of the view that the complex nature of the health system promotes an environment where errors that occur in a clinical process may influence patients to accept inappropriate treatment and care from HCPs as an acceptable norm. Therefore, what may be needed is the development of a “trusting and trusted health system – which is not only seen as a producer of health or health care – but as a purveyor of a wider set of societal values and norms” (p. 1461) [49]. The author further points out that the health system can contribute to achieving these values and norms when its interaction with society is based on trust.

A very disturbing finding was the expression that was used by one participant who was of the view that HCPs regard patients as *“graves”* (dead bodies). Interestingly, this issue about patients being treated as “bodies” has been explored by Henderson [50] in her observational study of clinical encounters of nurses with patients in the intensive care unit. She said that the routine charting of patients’ progress in the clinical process “separates the body into physical components which can be measured…this knowledge has not only empowered particular kinds of practice, but has also invented a new patient…the recorded body – a body about which little is known at an emotional level but everything at a biochemical and physiological level…this practice therefore shapes the health care practice” (pp. 937–938). Based on our interpretation of our participants’ verbatim accounts, the focus on recording of seizures and medication adherence may inhibit treatment of patients as autonomous people who are able to deliberate [51]. Therefore, in order to reduce ill care of this group of patients, there is a need to educate HCPs on strategies that promote what Thomas and McDonagh (p. 4) [52] refer to as “empathetic modelling” and interventions that can develop patients and HCPs skills in interactive and critical health literacy [53].

### Limitations

Although we used different approaches in our recruitment strategy to ensure rigour, we may have also not included those who were not known by the local gate keepers. Our study findings were from a small sample in an urban Xhosa speaking township and cannot be generalized. However, they confirm, complement and expand on findings regarding poor access to appropriate treatment and care of PWE reported in other LMICs [3, 4] and in some studies done in Cape Town [23, 41]. To ensure rigor, MJK used local gate keepers in the recruitment process and combined snowball and purposive sampling methods and also used field assistants to locate homes of listed participants who were living in shack dwellings and would be difficult to locate due to structural problems.

## Conclusion

Our study described perceived difficulties affecting access to appropriate treatment and care of PWE who were interviewed for the study. The findings show that the participants have by their accounts in fact experienced perceived inappropriate treatment and care on their routine follow up clinical visits. We argue that these experiences could be due to the power and nature of the RTCS. Our data suggest a need for appropriate interventions to address health systems factors affecting treatment and care of this marginalized and vulnerable group of patients.

## Abbreviations

AEDs: anti-epileptic drugs
AIDS: acquired immune deficiency syndrome
ARV: antiretroviral treatment
CHC: Community Health Centre
COPDs: chronic obstructive pulmonary diseases
EMs: explanatory models
HCPs: health care practitioners
HIV: human immunodeficiency virus
LMICS: lower middle income countries
mhGAP: Mental Health Gap Action Program
NYDA: National Youth Development Agency
NCDs: non-communicable diseases
PWE: patients’ with epilepsy
PGWC: Provincial Government of the Western Cape
RTCS: routine task-centred system
TB: tuberculosis
THO: Traditional Healers Organisation
NGOs: Non-governmental Organisations
WHO: World Health Organization
